# Mechanism of action and neuroprotective role of nicorandil in ischemic stroke

**DOI:** 10.1016/j.heliyon.2024.e26640

**Published:** 2024-02-20

**Authors:** Maryam Owjfard, Negin Rahmani, Arashk Mallahzadeh, Mahnaz Bayat, Afshin Borhani-Haghighi, Farzaneh Karimi, Mohammad Reza Namavar

**Affiliations:** a Clinical Neurology Research Center, Shiraz University of Medical Sciences, Shiraz, Iran; b Behbahan Faculty of Medical Science, Behbahan, Iran; c Histomorphometry & Stereology Research Center, Shiraz University of Medical Sciences, Shiraz, Iran; d Department of Anatomical Sciences, School of Medicine, Shiraz University of Medical Sciences, Shiraz, Iran

**Keywords:** Ischemic stroke, Nicorandil, K^+^ channels, Nitric-oxide, Blood pressure

## Abstract

Nicorandil is a dual mechanism anti-anginal agent that acts as a nitric oxide (NO) donor and a potassium (K^+^) channel opener. Recent studies have evaluated the effect of nicorandil on ischemic stroke. Neurons have a low tolerance to hypoxia and therefore the brain tissue is significantly vulnerable to ischemia. Current approved treatments for ischemic stroke are tissue plasminogen activators and clot retrieval methods. The narrow therapeutic time window and lack of efficacy in restoring the dying neurons urge researchers to develop an alternative approach.

In the terminal stages of anoxia, K^+^ channels induce hyperpolarization in various types of neuronal cells, leading to decreased neuronal activity and the preservation of the brain's energy. Nicorandil can open these K^+^ channels and sustain the hyperpolarization phase, which may have a neuroprotective effect during hypoxia. Additionally, we review how nicorandil can improve overall stroke outcomes through its anti-inflammatory, anti-oxidative, and edema-reducing effects. One of the major components evaluated in stroke patients is blood pressure. Studies have demonstrated that the effect of nicorandil on blood pressure is related to both its K^+^ channel opening and NO donating mechanisms. Since both hypertension and hypotension need correction before stroke intervention, it's crucial to consider the role of nicorandil and its impact on blood pressure. Previously published studies indicate that the right dosage of nicorandil can improve cerebral blood flow without significant changes in hemodynamic profiles. In this review, we discuss how nicorandil may contribute to better stroke outcomes based on previously published literature and laboratory findings.

## Highlights

• K^+^ channels can be found in neurons and contribute to neuroprotection.
• K_ATP_ channels preserve the brain energy during ischemia.
• Nicorandil contributes to neuroprotection through diverse mechanisms.
• Nicorandil's effect on blood pressure is contributable to both its K^+^ channel opening and nitric oxide donating mechanism.
• Nicorandil improves cerebral circulation without affecting hemodynamic profiles.

## Graphical abstract

**Figure:**
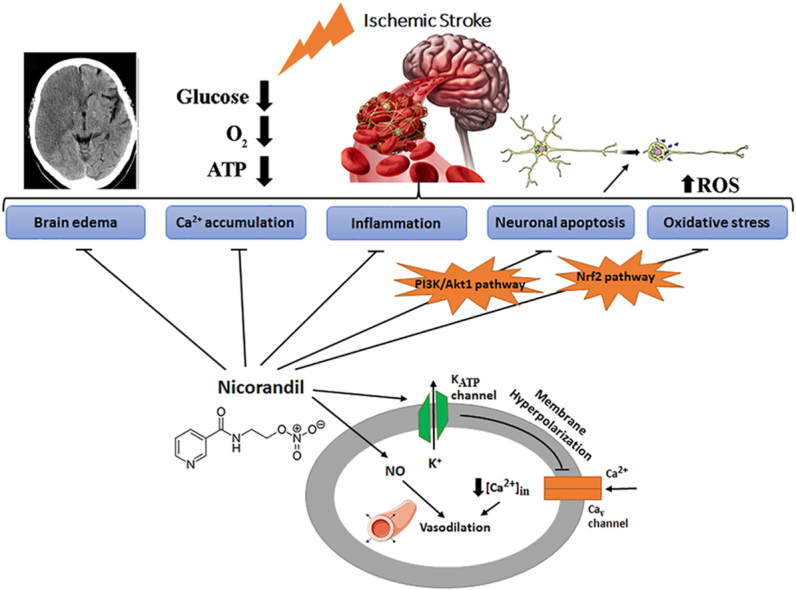

## 1 Introduction

Stroke is among the leading causes of morbidity and mortality worldwide [1]. It is a result of either sudden loss of blood perfusion to the brain known as ischemic stroke (IS) or a rupture of cerebral blood vessels causing intra-cerebral hemorrhage [2]. In an individual presenting with IS, there is an area in the brain that receives between 10% and 30% of its normal blood flow called the “penumbra” [3]. This tissue, unlike the ischemic core, has not undergone irreversible infarction yet, but the neurons are electrically malfunctioning and causing neurological deficits. The principle of treatment is to restore blood to the penumbra and prevent permanent neuron death.

To date, only tissue plasminogen activator (tPA) and mechanical thrombectomy are approved for restoring blood perfusion to the ischemic area [4,5]. Only a small percentage of individuals (maximum of 5%) are eligible to receive tPA therapy in the acute phase of IS, mainly due to delays in getting to the hospital or the narrow therapeutic time window [6]. The recanalization efficacy of intra-venous tPA is limited in IS [7]. Therefore, not only many patients do not meet the criteria for tPA therapy, but for a significant number of patients, the tPA therapy is ineffective. Mechanical thrombectomy is indicated for individuals experiencing acute ischemic stroke resulting from a significant blockage in a major artery within the brain's front region, provided that treatment can be initiated within 24 h from when their last known normal neurological state was observed. Two challenges may restrict the use of mechanical thrombectomy, Firstly, only around 10% of patients with acute ischemic stroke exhibit a substantial blockage in a major artery in the front part of the brain and present themselves for eligibility within a 6-h timeframe [[8], [9], [10]]. Secondly, only a limited number of stroke centers possess the necessary resources and specialized skills to provide this treatment [11]. That being said, due to the limitations in currently approved therapies for IS, it is crucial to investigate further treatments that could improve stroke outcomes for patients not eligible or who have failed these treatments.

The underlying mechanisms of ischemia for acute myocardial infarction (AMI) and acute IS are similar [12]. Nicorandil is a hybrid of an adenosine triphosphate (ATP)-sensitive potassium (K^+^) channel (K_ATP_) opener and nitrates. It can increase the outflow of K^+^ from cells, inhibit the inflow of calcium (Ca^2+^), reduce the Ca^2+^ overload, and consequently reduce the incidence of arrhythmia. It possesses the potential to expand the small coronary arteries and increase the coronary blood flow. These effects could be beneficial in the acute phase of IS [13]. Furthermore, animal studies have shown that nicorandil has therapeutic effects on various nervous system disorders, including its ability to protect against spinal cord issues, cerebral ischemia, and brain injuries by diminishing oxidative damage, mitigating inflammatory reactions, and employing anti-apoptotic mechanisms [[14], [15], [16]].

## 2 Nicorandil

Nicorandil is a nicotinamide nitrate with vasodilatory and K^+^ channel opening effects.

Its cardioprotective effects are attributable to its impacts on K_ATP_ channels which protect the ischemic tissue from losing ATP [[17], [18], [19]]. When consumed, nicorandil is primarily metabolized through oxidation to nicorandil-*N*-oxide and hydroxy nicorandil, and by denitration to *N*-(2-hydroxyethyl)-nicotin-amide. It is then transformed into nicotinamide, nicotinic acid, and *N*-methyl-nicotinamide. It has a half-life of 45 min during the first rapid phase of elimination after oral or intravenous administration [20]. Nicorandil acts as both a K_ATP_ channel opener and a nitric oxide (NO) donor [21].

## 3 Nicorandil and ischemic stroke

Ischemic conditions deprive the affected organ of the oxygen and nutrients needed for normal function. Neurons have a low tolerance to severe hypoxia resulting in cerebral anoxia [22]. The absence of oxygen and the resulting anoxia lead to the extracellular accumulation of glutamate, which can be excitotoxic [23]. It appears that anoxia-induced vulnerability can be related to an accompanied rise in intracellular Ca^2+^ concentration caused by increased glutamate concentrations in the interstitial space. Ca^2+^ elevations in such neurons are primarily attributed to influx through Ca^2+^-permeable glutamate receptors and voltage-activated Ca^2+^ channels during anaerobic depolarization [22,24,25]. Furthermore, inhibition of ATP-dependent ion pumps, such as the Na^+^/K^+^-ATPase, impairs Ca^2+^ extrusion and therefore, contributes to an excessive rise in Ca^2+^ [25]. The Ca^2+^ overload and ATP consumption by depolarized mitochondria lead to a cascade of molecular pathologies that ultimately result in cell death [23,26].

In a variety of neurons, a K^+^ channel induces hyperpolarization following the terminal anoxic depolarization [22,27]. This hyperpolarization leads to decreased neuronal activity and therefore, transmembrane ion fluxes which diminish the activity of ion pumps that absorb 50% of the brain's energy [28]. That being said, if the K^+^ channels persist in the hyperpolarization phase for a reasonable amount of time, it might have a protective effect during brain hypoxia [29]. It has been demonstrated that K_ATP_ channels mediate hypoxic hyperpolarization [30,31]. K_ATP_ channels are subtypes of K^+^ channels that conduct inward-rectifier potassium current in response to cellular energy depletion. They are members of the inwardly rectifying K^+^ (Kir) superfamily and are multimers of four pore-forming Kir6 subunits and four Sulfonyl-Urea Receptor (SUR) subunits [32]. K_ATP_ channels are present in both the cellular membrane and the inner mitochondrial membrane, and they have been identified in various tissues, including the kidney, pancreatic beta cells, muscles, heart, and the nervous system [32].

### 3.1 Cardiovascular K_ATP_ channels and protection from ischemic injury

Nicorandil attends effective therapy in AMI patients which has resulted in reducing the incidence of reperfusion injury and myocardial infarct size [33]. The opening of mitochondrial K_ATP_ channels leads to increased K+ entry into the mitochondria and inhibits the depolarization of the cell membrane, thereby preventing mitochondrial Ca^2+^ overload and protecting the heart [28]. Nicorandil also leads to vasodilation as a result of vascular smooth muscle cell hyperpolarization [34] that confers its vasodilator activity [28], causing a reduction in systemic vascular resistance, mean arterial pressure, pulmonary capillary wedge pressure, and left ventricular end-diastolic pressure. Additionally, nicorandil dilates large coronary arteries, decreases coronary vascular resistance, and increases coronary artery blood flow. According to a meta-analysis, treatment with nicorandil as an adjunct to reperfusion and percutaneous coronary intervention therapy improves heart functional outcomes after AMI by improving the microvascular function, reducing infarct size, and increasing the left ventricular ejection fraction [35]. This study demonstrated that nicorandil decreased all-cause mortality in coronary artery disease patients irrespective of the delivery route of this drug [35].

### 3.2 Cerebral K_ATP_ channels and possible protection from ischemic injury

In the brain, K_ATP_ channels are present in the substantia nigra, neocortex, hippocampus, and hypothalamus. These channels are highly expressed in interneurons and granule cells, which are resistant to ischemic injury [32]. A previous study has demonstrated that brain mitochondria contain six to seven times more mitochondrial K_ATP_ channels compared with the mitochondria in the heart tissue [36]. The expression of subunits differs across the brain. Neurons mostly express Kir6.2 and SUR1, whereas astrocytes express Kir6.1, SUR1, and SUR2 subunits [32].

The neuroprotective role of mitochondrial K_ATP_ channels in cerebral ischemia has been investigated [32]. It has been revealed that cortical neurons in Kir6.2 (−/−) K_ATP_ channel knockout mice are more vulnerable to ischemia compared with the wild-type neurons in a middle cerebral artery occlusion (MCAO) model [32]. Also, overexpression of the Kir6.2 leads to high resistance to ischemia and a reduction in ischemic brain damage in cortical neurons [37]. The tolerance to anoxia may be due to a long-term hyperpolarization mediated by K_ATP_ channels [29]. K_ATP_ channel openers have been shown to attenuate cortical neuronal death induced by oxygen-glucose deprivation (OGD) in rats [38]. Pre-conditioning of neural stem cells (NSC) using nicorandil has shown increased brain-derived neurotrophic factor (BDNF). BDNF is a neurotrophic factor that modifies neurogenesis and survival signaling pathways. It has also been observed that BDNF expression in preconditioned NSC led to activation of the phosphoinositide 3-kinases (PI3K) activity. PI3K is an enzyme involved in cellular functions that can lead to cell survival and simulate neurite growth [39,40]. This study also demonstrated the anti-inflammatory properties of nicorandil-preconditioned NSC, where the preconditioning could downregulate pro-inflammatory cytokines (IL-1β, IL-6, IL-12, and TNF-α) and increase the production of anti-inflammatory mediators (IL-10) [41].

Due to the restricted oxygen and nutrient delivery and the consequent decrease in the ATP/ADP ratio that happens during cerebral ischemia, K_ATP_ channels open and cause neuronal hyperpolarization. A decrease in the ionic pump activity reduces the metabolic needs of the neurons and protects them against excitotoxicity that occurs during anoxia [29,32]. Few *in vivo* and *in vitro* studies evaluated the efficacy of nicorandil as a K_ATP_ channel opener in treating stroke [14,15].

Nicorandil has the potential to mitigate the progression of cerebral ischemia by alleviating factors such as Ca^2+^ overload, brain edema, and oxidative stress, and by demonstrating anti-inflammatory and anti-apoptotic properties. We describe the possible treatment effects of nicorandil on IS.

### 3.3 Role of nicorandil in cerebral ischemia

#### 3.3.1 Reducing calcium overload

Nicorandil can protect against ischemic injury through diverse mechanisms. As described earlier, Ca^2+^ rise is one of the major causes of cell death. The dysfunction of the Na^+^/k^+^ ATPase pump as a result of ischemia occurs within minutes and leads to the accumulation of Ca^2+^ and activation of Ca^2+^-dependent enzymes [42]. The opening of mitochondrial K_ATP_ channels decreases mitochondrial Ca^2+^ overload. A decrease in mitochondrial Ca^2+^ inhibits the opening of the mitochondrial permeability transition pore, a transmembrane protein residing in the mitochondrial inner membrane. This pore opens during reperfusion and is responsible for mitochondrial swelling and initiation of mitochondria-dependent apoptosis [43]. Activation of the K_ATP_ channel can also decrease intracellular Ca^2+^ levels. Nicorandil has been shown to alleviate intracellular Ca^2+^ overload in a rat cardiomyocyte cell line when the cells underwent hypoxia/reoxygenation injury [44]. Another study also showed that nicorandil reduced intracellular Ca^2+^ accumulation induced by OGD in the rat hippocampal CA1 pyramidal cells [45].

#### 3.3.2 Reducing brain edema

IS leads to cerebral edema due to the disruption of the blood-brain barrier (BBB) during the first 24–48 h, and reaches a maximum extent of 3–5 days from the occurrence of IS [42]. The BBB disruption improperly permits serum proteins to shift from the blood to the brain parenchyma and lead to a type of vasogenic edema [46]. Ca^2+^ influx into the cells plays a key role in damaging the BBB [47]. K_ATP_ channel openers such as nicorandil can decrease intracellular Ca^2+^ which can help preserve the BBB integrity [45]. It has been observed that aquaporins 4 (AQP-4) (plasma membrane channels specialized for water transport) are involved in ischemic brain damage and brain edema [48]. The effect of nicorandil on cerebral injury following cardiac arrest was investigated and showed that AQP-4 expression in the brain tissue of pigs was significantly lower in the nicorandil-treated group than in the control group 6 h after the return of systemic circulation. The decreased expression of AQP-4 induced by nicorandil might translate into a reduction in the degree of brain edema after ischemia [49]. In a rat MCAO model, nicorandil administration for 3 days reduced cerebral edema, which was determined by measuring the brain water content [14].

#### 3.3.3 Anti-oxidative effect

Ischemia induces the production of reactive oxygen species that can damage cellular components [42]. In an *in vitro* study on astrocytes, nicorandil treatment decreased the expression of endoplasmic reticulum stress-related proteins in response to OGD [50]. Nicorandil has demonstrated neuroprotective effects against H_2_O_2_-induced neuronal stress [51] and inhibited the production of reactive oxygen species in endothelial cells under oxidative stress [52]. In a mouse model of 2-vessel occlusion, nicorandil administration has shown a decrease in oxidative stress markers such as catalase and superoxide dismutase in the brain [53].

#### 3.3.4 Reducing inflammation

Increased infiltration of peripheral leukocytes into the brain has been observed hours to days after ischemic injury [42]. Activated microglia, which are the brain's resident inflammatory cells, become phagocytic and migrate to the ischemic core. This triggers an immune response and release of interleukins such as IL-1 β, tumor necrosis factor (TNF), and IL-6 [54]. The pro-inflammatory cytokines that have been activated after IS disrupt the BBB which results in further infiltration of inflammatory cells [55,56]. Release of the chemokine matrix metalloproteinases (MMP), particularly MMP-9, by the innate immune system results in further disruption of the BBB leading to edema and growth of the infarct area [57] Downregulation of immune components has been found to contribute to better stroke outcomes [58]. K_ATP_ channels are likely involved in regulating the production of IL-1β by microglia after OGD. The pre-treatment of the microglial cell line with nicorandil can decrease the production of IL-1β [59]. Treatment with nicorandil has been shown to attenuate the inflammatory response induced by OGD [50] and decrease inflammatory markers in the brain after cerebral ischemia [53]. Nicorandil has also been shown to inhibit platelet activation, probably by increasing the activity of endothelial NO [52]. In a swine cardiac arrest model evaluating the effect of nicorandil on the resulting cerebral injury, TNF-α and IL-6 levels were significantly lower in the nicorandil group than in the control group after three and 6 h of the return of systemic circulation [16]. In a clinical study on 157 patients with unstable angina who received nicorandil in addition to the conventional treatment, high-sensitivity *C*-reactive protein and MMP-9 were significantly decreased 21 days after nicorandil treatment compared with the control group, who merely received the conventional treatment [60].

#### 3.3.5 Anti-apoptosis

Apoptosis within the ischemic penumbra may occur after a substantial time delay [42]. In an *in vitro* study, nicorandil has been shown to protect cultured glioblastoma cells against H_2_O_2_-induced cell death [51]. Nicorandil also inhibited apoptosis induced by oxidative stress as evidenced by the suppression of the activity of caspase-3 in cerebellar granule neurons [61] and a neuroblastoma cell line in a cellular model of Alzheimer's disease [62]. Another study showed nicorandil could cause more neuronal density and less neuronal death in the face of ischemia [63] and that the effect of nicorandil was dose-dependent, as mice treated with higher doses of nicorandil (10 mg/kg and 20 mg/kg) showed a more considerable reduction in the number of TUNEL-positive cells than those treated with a low dose (5 mg/kg) [63]. They concluded that nicorandil has a neuroprotective role in ischemia-reperfusion (I/R) injury induced by deep hypothermic low flow by inhibiting apoptosis through the activation of the PI3K/Akt1 signaling pathway [63].

## 4 Effects of nicorandil in animal studies

The oral administration of 2 and 4 mg/kg of nicorandil once a day from the 7th day post-stroke until the 30th day caused a significant decrease in the infarct size and serum nitrosative stress, brain oxidative stress, cholinergic dysfunction, and inflammation in a mouse model of chronic cerebral hypoperfusion [53]. In a mouse model of brain I/R injury, the effects of nicorandil injection 15 min before the common carotid arteries occlusion were dose-dependent; a moderate number of pyknotic or shrunken nuclei were found in mice treated with lower doses of nicorandil (5 mg/kg), while pyknotic or shrunken nuclei were rarely found in mice treated with higher doses of nicorandil (10 mg/kg and 20 mg/kg) [63]. An injection of 10 mg/kg of nicorandil 30 min before MCAO resulted in a significant reduction in cerebral infarct volume [64]. In another study 5 mg/kg oral nicorandil administration once a day for three consecutive days after ischemia did not have a significant effect on neurobehavioral functions, count of neurons, non-neurons, and dead neurons, although it significantly reduced striatal infarct volumes [14]. In other investigations, normal and diabetic Wistar rats were given intraperitoneal nicorandil (5 mg/kg) for three days. They found a significant reduction in neurobehavioral impairments, cerebral infarct volume, and caspase enzyme levels compared to I/R control and I/R diabetic animals without affecting the animals' blood pressure (BP) [51]. Another study also reported that 5 mg/kg oral nicorandil administration once a day for three consecutive days after ischemia significantly decreased the neurological deficits and density of neuronal neighbors. It could not preserve the regular spatial distributions of M1 cortical neurons after MCAO. It also could not significantly improve motor function or reduce the ischemic lesion size [15]. Based on the mentioned preclinical studies, the effects of nicorandil on stroke outcomes can vary depending on the time of administration (see Table 1). Therefore, one major factor that needs attention is the temporal effects of nicorandil in stroke management.

**Table 1:** Animal experiments with nicorandil in stroke.

References	Animal model	Type of ischemia	Route of administration	Time of administration	Dose	Evaluated parameters	Main findings
[64]	male Wistar rats	MCAO (2 h)	Intraperitoneal injection	30 min before MCAO	10 mg/kg	• Infarct size	▶ CTRL > NIC
						• Content of MDA	▶ CTRL > NIC
						Expression of SDH and CO in the brain	▶ CTRL < NIC
[14]	Sprague–Dawley rats	MCAO (60 min)	oral gavage	for 3 consecutive days after MCAO	5 mg/kg	• Neurobehavioral function	▶ CTRL ∼ NIC
						• BBB disruption	▶ CTRL ∼ NIC
						• Brain water content	▶ CTRL ∼ NIC
						• Infarct size	▶Striatal infarction volume: CTRL > NIC
						• The number of neurons, non-neurons, and dead neurons in the right cortex	▶ CTRL ∼ NIC
						• The expression levels of NF-kB and Nrf2.	▶ NF-kB: CTRL < NIC ▶ Nrf2: CTRL < NIC
Khastkhodaei et al. (unpublished)	male Sprague–Dawley rats	MCAO (60 min)	oral gavage	30 min after MCAO	1 (NIC_1_) and 3 (NIC_3_) mg/kg	• Neurological Assessment	▶ CTRL > NIC_1_> NIC_3_
						• Stereological evaluations	▶ Infarct volume & Dead neuron number: CTRL > NIC_1_> NIC_3_ ▶ Number of cortical neurons and non-neurons: CTRL < NIC_1_< NIC_3_
						• Voronoi tessellations	▶ NIC preserves the pattern of neuronal distribution in its normal, i.e. regular pattern.
[53]	male albino mice	Bilateral Common Carotid Artery Ligation or 2-Vessel Occlusion	oral gavage	once daily for 24 days	2 and 4 mg/kg	• Cognitive impairment (Morris water maze)	▶ CTRL > NIC
						• Infarct size	▶ CTRL > NIC
						• Assessment of serum Nitrite/Nitrate level	▶ CTRL **>** NIC
						• Brain AChE, BLP, and MPO level • Brain GSH, SOD, CAT level	▶ CTRL **>** NIC ▶ CTRL > NIC
[63]	Wild-type C57BL/6 mice	Deep hypothermic low flow (DHLF)	Intraperitoneal injection	15 min before occlusion of the CCAs	5 mg/kg, 10 mg/kg, or 20 mg/kg)	• Number of apoptotic & necrotic cells in the hippocampus • pyknotic nuclei	▶ CTRL > NIC ▶ CTRL < NIC
						Western blot analysis • Bcl-2 levels Bax levels •	▶ CTRL < NIC ▶ CTRL > NIC
						• PI3K/Akt signaling pathway: phosphorylation status of Akt (*p*-Akt)	▶ CTRL < NIC ▶NIC activates the PI3K/Akt signaling pathway
[51]	male Wistar rats	MCAO (2 h)	Intraperitoneal injection	for 3 consecutive days after MCAO	5 mg/kg	• Neurobehavioral function	▶ CTRL < NIC
						• Infarct size	▶ CTRL > NIC
						• Caspase-3 levels of brain tissue	▶ CTRL > NIC
						• Blood pressure	▶ CTRL ∼ NIC
[15]	male Sprague–Dawley rats	MCAO (60 min)	oral gavage	for 3 consecutive days after MCAO	5 mg/kg	• Neurological deficits score	▶ CTRL > NIC
						• Rotarod and wire hanging performance	▶ CTRL ∼ NIC
						• Infarct Volume	▶ CTRL ∼ NIC
						• Histology (stained with cresyl violet; Voronoi polygon area, density of neighbors, and spatial distribution of primary motor cortical neurons)	▶Voronoi Polygon area: CTRL < NIC ▶Voronoi Polygon density: CTRL > NIC ▶spatial distribution: CTRL and NIC: Random pattern Sham: Regular pattern

**Abbreviations:** AChE, Acetylcholinesterase; BLP, Brain lipid peroxidation; CAT, Catalase; CBF, Cerebral blood flow; CCA, Common carotid artery; CO, Cytochrome oxidase; CTRL, Control; GSH, Glutathione; MDA, Malondialdehyde; MPO, Myeloperoxidase; NIC, Nicorandil; SDH, Succinate dehydrogenase; SOD, Superoxide dismutase.

## 5 Therapeutic implications of nicorandil in ischemic stroke

The therapeutic implications and potential conjunct treatment of nicorandil in the context of ischemic injury are significant due to its dual mechanism of action as a NO donor and a K^+^ channel opener [21]. Given the similarity between ischemic injury in the heart and brain, nicorandil's potential to enhance cerebral blood flow (CBF) and reduce ischemic damage may have implications for stroke treatment [12]. Nicorandil may be beneficial as an adjunct therapy to complement standard stroke treatments. When administered alongside thrombolytic medications, which dissolve blood clots, nicorandil could help enhance the efficacy of clot-dissolving treatments [65]. Furthermore, nicorandil's potential to reduce inflammation, oxidative stress, and apoptosis could be considered alongside other neuroprotective strategies to minimize brain damage and improve outcomes [14]. In cases where reperfusion (restoration of blood flow) is a primary treatment goal, nicorandil's vasodilatory properties may aid in achieving optimal blood flow restoration in ischemic tissues and also reduce reperfusion injury through K_ATP_ channels [66].

## 6 Nicorandil effects on blood pressure

Hypertension is one of the modifiable risk factors for IS. Optimal blood pressure (BP) management differs in stroke patients. The 2018 guideline of the American Heart Association recommends patients eligible for tPA therapy should have their systolic BP below 185 mmHg and their diastolic BP below 110 mmHg [67]. However, hypotension is just as important as hypertension in stroke management. When cerebral ischemia occurs, studies have shown BP elevates even in patients who haven't had a history of hypertension [68]. This physiologic rise might be beneficial by improving CBF in the ischemic area [69]. A meta-regression analysis demonstrated that a mild reduction in BP may reduce mortality, however, significant falls or increases in BP are associated with worse clinical outcomes [70].

Nicorandil is a nitrate in chemical structure and causes vascular smooth muscle relaxation by increasing intracellular cyclic guanosine monophosphate (cGMP) which leads to NO release [28,71]. Nitrates like nicorandil cause peripheral venous dilatation leading to a reduction in preload and, therefore, coronary artery dilatation and lowering of systemic BP [28]. Nicorandil has a dual mechanism of action which can also act as a K^+^ channel opener that isn't related to its chemical structure [58]. Studies have demonstrated that non-nitrate K^+^ channel openers like BRL 34915 and pinacidil decrease systemic BP [72]. Unlike nitrates, nicorandil improves endothelial dysfunction [34]. Many studies have suggested that nicorandil has additional actions as an arterial K_ATP_ channel agonist, resulting in more “balanced” arterial and venous vasodilatation than nitrates [34] and the minimum effective doses of nicorandil produce its antianginal effects with a little hemodynamic side effect on BP, heart rate, or cardiac contractility [73].

A study that evaluated the effect of nicorandil on BP and CBF showed that nicorandil has a dual effect and is dose-dependent. The administration of 1 mg/kg nicorandil increased CBF (11.6 ± 3.6 vs. 0.5 ± 0.7%) in intact mice without reducing heart rate or systemic BP, while higher doses (5–10 mg/kg) resulted in a decrease in CBF (−6.6 ± 8.2 vs. 0.1 ± 1.1%), systemic BP (62 ± 12 vs. 76 ± 6 mmHg) and increased heart rate (523 ± 32 vs. 466 ± 18 beats/min) [74]. They concluded that the clinical dose of nicorandil can increase CBF without affecting systemic hemodynamics. Furthermore, intraperitoneal administration of nicorandil (5 mg/kg) for 3 days in a rat model of MCAO decreased neurobehavioral deficits, cerebral infarct volume, and caspase enzyme levels compared to Ischemic/Reperfusion control animals with no effect on BP (90 vs. 97 mmHg) [51].

Clinical trials on human subjects show nicorandil has a beneficial impact on mortality and morbidity in coronary artery disease patients [75] and post-AMI patients [76]. Previous studies showed that intracoronary injection of 4 mg/h for 24 h [77], intravenous of 12 mg [78], and 6 mg [79] nicorandil bolus exerted no significant effect on BP in ST-segment elevation myocardial infarction patients. While a bolus of 5 mg nicorandil, followed by 2 mg/h or 4 mg/h for 12 h continuously slightly decreased BP [80]. Supine BP was decreased equally and significantly after a single acute dose of 10, 20, and 30 mg nicorandil, with a peak after 4–6 h (10 mg: 21/-8 mmHg; 20 mg: 20/-9 mmHg; 30 mg: 29/-17 mmHg), and its hypotensive effect diminished after 24 h without a change in the heart rate [81]. In another study, nicorandil (2 or 4 mg/h) was given over 12 h after the percutaneous coronary intervention; systolic BP and systemic vascular resistance index were significantly reduced, suggesting that after a successful percutaneous coronary intervention, nicorandil reduced an afterload in patients with stable angina pectoris [82]. Therefore, these studies supported that a dose under 10 mg of intravenous nicorandil produced no deleterious effect on BP, but continuous intravenous administration of nicorandil led to a decrease in BP. The typical starting dose of nicorandil is 5–10 mg twice daily and can be up-titrated to 20 mg or a maximum of 30 mg twice daily [83]. Also, the cardioprotective dose of this drug utilized in animal models of I/R injury is considerably higher than the dose used in humans.

The use of nicorandil in the early post-stroke period may be effective in improving cerebral dysautoregulation in patients with hypertension. A study has demonstrated that nicorandil administration exerts beneficial effects on cerebral circulation in patients with chronic cerebral infarction by reducing mean arterial BP with increasing regional CBF [84]. It should be mentioned that the dose of nicorandil used should be able to increase CBF without affecting BP, intracranial pressures, or impairing cerebral autoregulation. A summary of these findings is presented in Table 2.

**Table 2:** Clinical trials for the effects of nicorandil on blood pressure.

References	Type of injury	Route of administration	Time of administration	Dose	Evaluated parameter	Main findings
[77]	Patients with STEMI	Intracoronary	24 h	4 mg/h	• Systolic BP	▶ No difference was observed before and after PPCI
					• Diastolic BP	▶No difference was observed before and after PPCI
[85]	Patients with STEMI	Intravenous	20–30 min before the PCI	12 mg	• BP	▶ CTRL = NIC
[79]	Patients with STEMI	Intracoronary	After PCI	6 mg	• BP	▶ CTRL = NIC
[80]	Patients with stable angina pectoris	Intravenous	12 h after PCI	2–4 mg/h	• BP	▶ CTRL > NIC
[86]	Hypertensive patients	Intravenous	9 a.m. for 3 days	10,20,30 mg	• Supine BP	▶10 mg: 21/-8 mmHg; 20 mg: 20/-9 mmHg; 30 mg: 29/-17 mmHg
[87]	Patients with stable angina	Intravenous	After PCI	5 mg bolus followed by 2 or 4 mg/h for 12 h	• BP	▶ CTRL > NIC

**Abb Abbreviations:** AMI, Acute myocardial infarction; BP, Blood pressure; CTRL, Control group; IHD, Ischemic heart disease; NIC, Nicorandil treated group; PCI, Percutaneous coronary intervention; PPCI, Primary percutaneous coronary intervention; STEMI; ST-elevation myocardial infarction.

## 7 Conclusion

In this review, we have provided evidence that K_ATP_ channels not only have neuroprotective effects through preserving the brain's energy but also through modulating the immune response following IS. Nicorandil is a K^+^ channel opener and NO donor. Opening of mitochondrial K_ATP_ channels by nicorandil can protect the brain penumbra during ischemia by reducing intracellular Ca^2+^ levels and preventing apoptosis and cell death. The K^+^ channels induce hyperpolarization and can decrease neuronal activity and therefore, preserve the brain's energy.

Nicorandil has demonstrated encouraging outcomes in reducing inflammation, cerebral edema, and preventing apoptosis, factors that can have a substantial impact on the outcomes of stroke. These findings which support that nicorandil may improve overall stroke outcomes are mainly from animal studies, therefore further trials are needed to better evaluate its efficacy in IS.

Nicorandil's effects on BP need further attention in stroke management. Based on previous studies, it seems that the right dose of nicorandil can improve CBF without affecting systemic hemodynamics. This is due to the NO pathway and K^+^ channel opening mechanism. Significant falls in blood pressure can lead to worse stroke outcomes as it can disrupt blood perfusion. Therefore, it's crucial to consider nicorandil's role in this matter and choose the appropriate dosage for stroke management. However, the use of nicorandil in managing stroke is not yet a standard practice and further trials are needed to establish its safety and effectiveness in this context.

These findings bring new insights into how nicorandil can potentially have neuroprotective effects and promising outcomes that may change future prognosis and IS management.

## Funding

This research did not receive any specific grant from funding agencies in the public, commercial, or not-for-profit sectors.

## Data availability

No data was used for the research described in the article.

## CRediT authorship contribution statement

**Maryam Owjfard:** Writing – review & editing, Writing – original draft, Software, Methodology, Investigation, Formal analysis, Data curation, Conceptualization. **Negin Rahmani:** Writing – review & editing, Writing – original draft. **Arashk Mallahzadeh:** Writing – review & editing, Writing – original draft. **Mahnaz Bayat:** Writing – review & editing, Writing – original draft, Formal analysis. **Afshin Borhani-Haghighi:** Writing – review & editing, Writing – original draft, Data curation. **Farzaneh Karimi:** Writing – review & editing, Writing – original draft, Methodology. **Mohammad Reza Namavar:** Writing – review & editing, Visualization, Validation, Supervision, Project administration, Methodology, Data curation, Conceptualization.

## Declaration of competing interest

The authors declare the following financial interests/personal relationships which may be considered as potential competing interests: The authors declare that they have no competing interests. This research did not receive any specific grant from funding agencies in the public, commercial, or not-for-profit sectors. If there are other authors, they declare that they have no known competing financial interests or personal relationships that could have appeared to influence the work reported in this paper.
